# Hand hygiene behavior among Sri Lankan medical students during COVID-19 pandemic

**DOI:** 10.1186/s12909-021-02783-9

**Published:** 2021-06-08

**Authors:** Guwani Liyanage, Madushika Dewasurendra, Ashan Athapathu, Lakmini Magodarathne

**Affiliations:** 1 Department of Pediatrics, Faculty of Medical Sciences, University of Sri Jayewardenepura, Nugegoda, Sri Lanka; 2 Newcastle University of Medicine, Johor, Malaysia; 3 Public Health Complex, Ministry of Health & Indigenous Medicine, Colombo, Sri Lanka

**Keywords:** Hand Hygiene, Pandemics, Students, Medical, Sri Lanka, Knowledge, Attitude, Behavior

## Abstract

**Background:**

Poor compliance with hand hygiene practices among medical students poses a risk for cross-infection. It has become more critical during the COVID-19 pandemic than ever before. This study aimed to determine the knowledge, attitudes, practices of hand hygiene among final-year medical students. It also explored reported hand hygiene behavior before the COVID-19 pandemic and the need for educational strategies to correct the deficiencies.

**Methods:**

A concurrent mixed-method approach was used. In the quantitative strand, a cross-sectional online survey was carried out via a Google form. Mann-Whitney U test and Chi-squared test were used for comparisons. In the qualitative strand, twelve participants were interviewed, based on a semi-structured interview guide and audio recorded. Transcribed data were evaluated with thematic content analysis.

**Results:**

A total of 225 final-year medical students were studied in the quantitative strand. Most were females. The mean score for knowledge was 3.35 ± 0.795 out of six. Of them, 31.6 % of participants scored below 3 points (< 50 % of the total). Most (78.9 %) had positive attitudes (score of > 80 %). Only 36.4 % reported “adequate” hand hygiene performance in all eight dimensions of the behavior domain. Noticeably, fewer participants reported to clean their hands after checking blood pressure (55.6 %), and only 66.2 % stated carrying a hand sanitizer in their pocket. Significant correlations were not found between reported behavior and attitudes (*p* = 0.821) or knowledge (*p* = 0.794). The qualitative strand with 12 respondents revealed the positive influence of both hierarchical and non-hierarchal role models. Time constraints, skin irritation, and workload pressures were the main barriers. Frequent reminders, supervision, and interactive teaching were suggested as methods to improve hand hygiene compliance. They also stated that increased enthusiasm was noted on hand hygiene during the COVID-19 pandemic compared to the pre-pandemic period.

**Conclusions:**

Most of the participants had positive attitudes towards hand hygiene. Yet, a considerable gap between attitudes and knowledge and reported hand hygiene behavior was evident. Coupling educational programs that use cognitive and behavioral methods, including role modeling, supervision, and frequent reminders, is recommended to bridge the knowledge-attitude-behavior gap.

**Supplementary Information:**

The online version contains supplementary material available at 10.1186/s12909-021-02783-9.

## Background

Hand hygiene, either by hand washing or chemical hand disinfection, remains the most critical measure to prevent nosocomial infections [1, 2]. With the pandemic of COVID-19, compliance with hand hygiene has become more important than ever before. Despite it being a simple procedure, many studies have shown that compliance is low among junior doctors and medical undergraduates [3, 4]. Specifically, the importance of this simple procedure is not sufficiently recognized despite the reduction of cross-infection by 55 % [5]. Continuous efforts are being made to ascertain effective and sustainable strategies to overcome poor compliance.

On 10th March 2020, the first Sri Lankan local national was tested positive for COVID-19 [6]. Following a lengthy island-wide lockdown for more than three months, schools and universities were re-opened in stages. One month after resuming the clinical placements, this survey was conducted to assess knowledge, attitudes, and reported behavior on hand hygiene measures among final-year medical students. This study also explored the reported hand hygiene behavior that prevailed before the COIVD-19 pandemic and the need for educational strategies.

## Methods

### Study design

A concurrent mixed-method design was used to achieve the objectives of this study. It was conducted in August 2020, one month after the commencement of clinical training of medical students following a prolonged university closure. At the time of data collection, the total number of reported COVID-19 cases was 2988, with 11 deaths in Sri Lanka, with a population of 21,833,000 [7]. Sample size calculations were done after considering the total number of final-year medical students (1350). The target sample size for the quantitative strand was 223 medical students in the final year with a ± 5 % margin of error, a confidence level of 95 % based on a recent study (77.7 % was hypothesized as the frequency outcome for hand hygiene practices for the population) [8]. Twelve medical students were interviewed in the qualitative strand. Ethical clearance was obtained from the Institutional Review Board of the Colombo South Teaching Hospital (CSTH/ERC/877) before the commencement of the study.

### Quantitative strand

As systematic sampling was not possible, the potential participants were sent invitations through social media (Viber/WhatsApp) with a link to an online Google form. The questionnaire accompanied the information sheet before providing a link to the questionnaire.

The questionnaire was designed to capture data on socio-demography, knowledge, attitudes, reported behavior, and practices among medical students, based on the World Health Organization (WHO) checklist for hand hygiene, a previously published questionnaire, and expert opinion on the content validity of questions [1, 3]. For knowledge assessment, six main multiple-choice questions were included. Four out of six questions had sub-categories. Each main question was given equal weightage and awarded one point. For questions with multiple categories, the mark was divided between numbers of sub-categories. If incorrect or marked as “do not know,“ the response was awarded zero points. The maximum score was 6 points.

There were four questions for assessing perception and attitudes on a 5-stage Likert scale (Strongly agree = 1, Agree = 2, Undecided = 3, Disagree = 4, Strongly disagree = 5). Total scores ranged from 5 to 20. Higher scores indicated better attitudes. It was assessed for internal reliability, using Cronbach’s α (0.826). There were eight items for assessing reported behavior related to hand hygiene on a 4-stage Likert scale (Always = 4, Often = 3, Sometimes = 2, Never = 1), a Cronbach’s α value of 0.814. Each question scored a maximum of four, and the total score ranged from four to 32. Higher scores indicated better hand hygiene behavior.

### Qualitative strand

After obtaining written informed consent, the qualitative data collection was carried out, adhering to the general COVID-19 guidelines, prevailing at the time of data collection. The convenience sampling method was used for recruitment. Medical undergraduates from clinical placements in one training unit were identified. Face-to-face interviews were carried out on twelve students. A structured interview guide was used. It explored several aspects related to hand hygiene (Table 1). Data anonymity and security were assured to the participants. The duration of the interview was not fixed. All interviews were led by a single interviewer (MD). All were audio-recorded and fully transcribed.

**Table 1:** Interview guide

*Can you tell the importance of hand hygiene practices? *
*Can you describe the influence of others on hand hygiene? What is your opinion on peers, doctors, patients, and nurses influencing your hand hygiene practices? Are there any role models?*
*Can you describe the reasons or barriers for others not adhering to hand hygiene practices? (Organizational and individual level). What is your experience with that?*
*Can you comment on the hand hygiene behavior at present, in the middle of a pandemic? Can you speak about hand hygiene behavior before the pandemic?*
*Can you describe ways of improving hand hygiene behavior among medical students?*

### Statistical data analysis

Different investigators evaluated the quantitative and qualitative data. Quantitative data were analyzed using the Statistical Package for Social Sciences (SPSS) software version 22. Data were checked for skewness and kurtosis. Categorical variables were expressed as numbers and percentages, and continuous variables as mean, median, and range. Since there are no validated cut-off values in the literature, we considered the following cut-off to categorize each domain. If the participants had indicated Likert scale option 4 (disagree) or 5 (strongly disagree) for all four questions in the attitude domain, he or she was considered as having “positive” attitudes towards hand hygiene. In the behavior dimension, the scores of “often” and “always” added to label the reported behavior as adequate. The knowledge score between males and females was compared with Pearson’s Chi-squared test. Comparison between each attitude dimension and knowledge (< 50 % and ≥ 50 %) was performed using Mann Whitney U test. Likewise, the connection of each behavior dimension with knowledge (< 50 % and ≥ 50 %) and attitudes (positive vs. negative) was explored. Univariate logistic regression analysis was used to identify the impact of attitudes and knowledge (continuous independent variables) on reported hand hygiene compliance (categorical dependent variable). Linearity between dependent and independent variables was confirmed with Box-Tidwell (1962) procedure.

Thematic content analysis was done on the transcribed audio records, in individual word files, of the qualitative survey. Two of the researchers (GL and MD) were involved in the manual sorting of qualitative data. Initially, we familiarized ourselves with the transcribed data. After that, each segment of data was coded with an open coding system. Each researcher did the initial coding of each transcript separately and then discussed and modified the coding framework after re-reading the transcripts. Subsequently, preliminary, descriptive themes were identified. Those themes were discussed and reviewed in the context of the whole data set. Final themes were established through the consensus of the two researchers.

## Results

### Quantitative strand

A total of 225 participants responded to the questionnaire. The majority were females (71 %). The mean age (± SD) was 26.7 ± 1.149 years.

#### Knowledge of hand hygiene practices

**Fig. 1:**
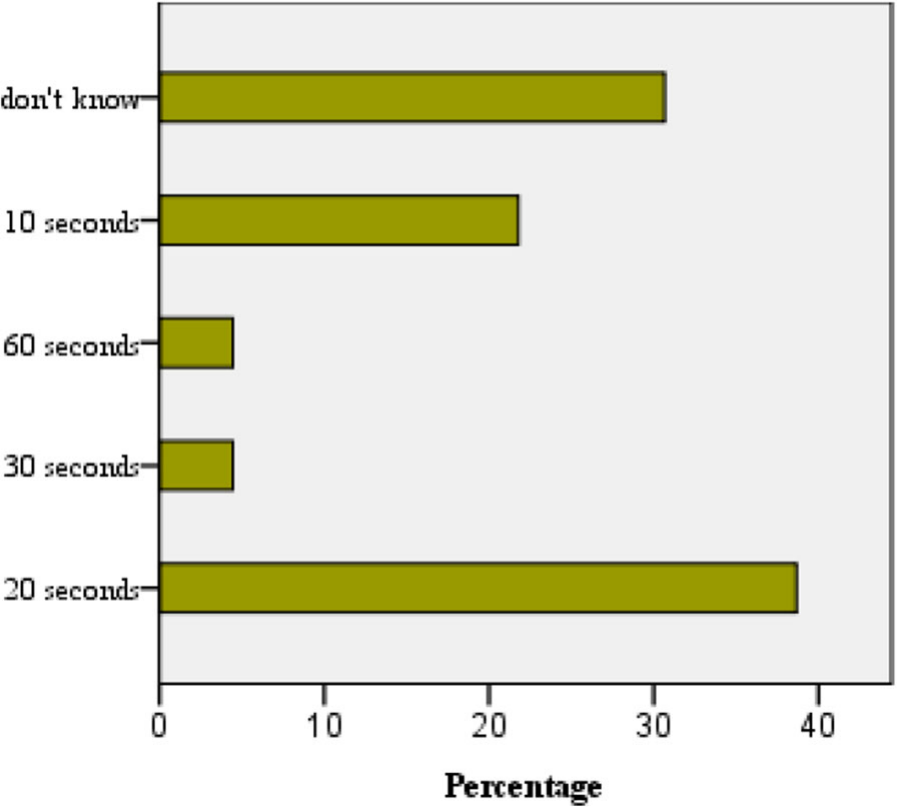
Responses to correct duration of required for hand hygiene with alcohol-based hand rub

The mean score for knowledge was 3.35 ± 0.795 out of six. Of them, 31.6 % of participants scored below 3 points (< 50 % of the total), and 80.4 % scored below 4 points. Also, scoring was relatively low on several knowledge dimensions. Notably, less than half knew the correct rubbing time and amount of hand rub to be used. Interestingly, 31 % did not know the suitable alcohol concentration in hand rub (Table 2; Fig. 1). Most knew alcohol hand rub is less effective than handwashing. They correctly identified the risk factors for organism colonization. Besides, most (73.8 %) misjudged hand cream used as a risk factor for colonization. Overall, the knowledge score was not different between males and females (*X*^2^ = 0.024, df = 1, *p* = 0.877). Gender-based comparisons of each knowledge dimension are depicted in Table 2.

**Table 2:** Comparison of knowledge in males and females

Questions on knowledge	All participants (*n* = 225)	Females (*n* = 160)	Males (*n* = 65)	*P*-value
Q1. What is the minimum time needed for scrubbing during handwashing with soap and water to kill most micro-organisms?	123 (54.7)	88 (55)	35 (53.8)	0.875
Q2. Minimum time needed for alcohol-based hand rub to kill most of the micro-organisms	87 (38.6)	62 (38.8)	25 (38.5)	0.968
Q3. Which of the following are true about alcohol hand rub and hand washing				
*Hand washing and hand rubbing are recommended to be performed in sequence (no)*	114 (50.7)	79 (49.4)	35 (53.8)	0.542
*Alcohol hand rub is more effective than handwashing (no)*	179 (79.5)	135 (84.4)	44 (67.7)	**0.005**
Q4. Which of the following should be avoided to prevent the increased likelihood of colonization of hands?				
*Wearing jewelry (yes)*	206 (91.6)	145 (90.6)	61 (93.8)	0.431
*Damaged skin (yes)*	200 (88.8)	145 (90.6)	55 (84.6)	0.194
*Regular use of hand cream (no)*	59 (26.2)	45(28.1)	14 (21.5)	0.309
Q5. Which of the following statements on alcohol-based hand rubs are true?				
*Apply a palmful of alcohol-based hand rub in the cupped hand*	84 (37.3)	54 (33.8)	30 (46.2)	0.081
*Rub only on the palmar surface of the hand (no)*	206 (91.6)	146 (91.3)	60 (92.3)	0.796
*Rub hands until dry (yes)*	109 (48.4)	78 (48.8)	31 (47.7)	0.886
*Alcohol solutions containing 60-80 % alcohol have* *efficacious microbicidal activity (yes)*	155 (68.9)	110 (68.8)	45 (69.2)	0.944
Q6. What are the instances where hand hygiene with alcohol rub is adequate without cleaning with soap and water?				
*Before palpation of the abdomen (yes)*	202 (89.7)	142 (88.8)	60 (92.3)	0.425
*Before giving an injection (yes)*	87 (38.6)	64 (40.0)	23 (35.4)	0.519
*After removing examination gloves (yes)*	92 (40.8)	61 (38.1)	31 (47.7)	0.186
*After visible exposure to blood (no)*	205 (91.1)	145(90.6)	60 (92.3)	0.829
*After checking blood pressure (yes)*	209 (92.8)	149(93.1)	60 (92.3)	0.688

Pearson’s chi-squared test was used for comparisons

#### Attitudes on hand hygiene practices

**Fig. 2:**
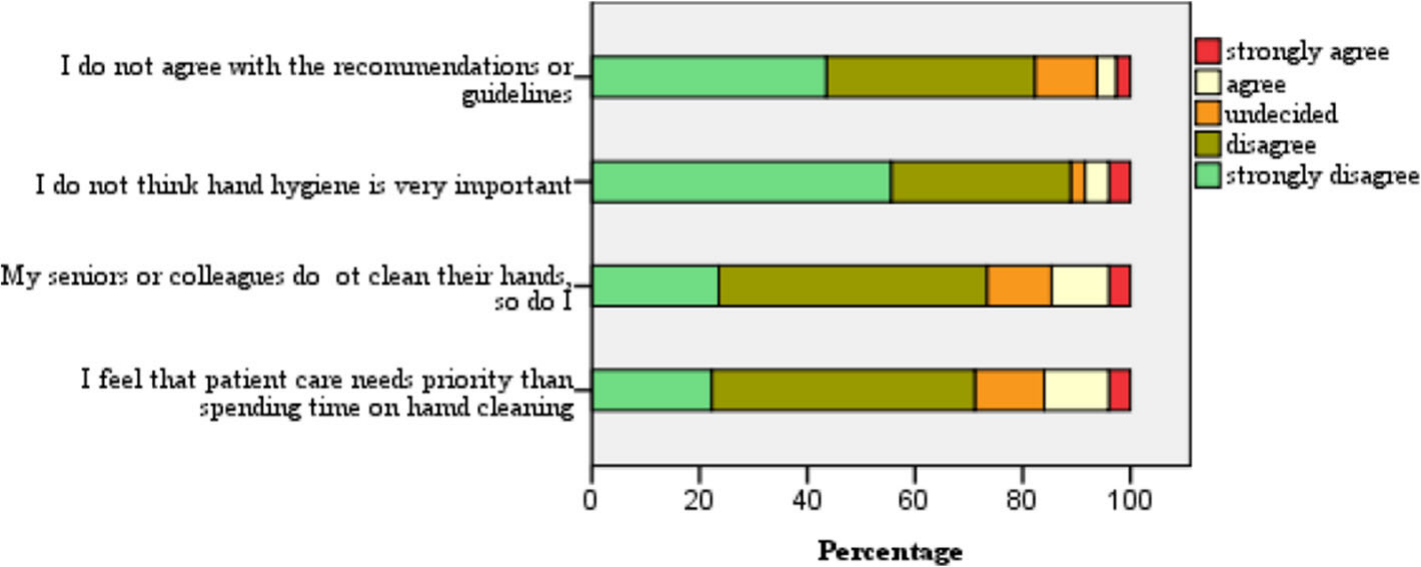
Participants’ attitude towards hand hygiene

The median score for attitude was 16 (range 4–20). Most (78.9 %) had positive attitudes (score of ≥ 80 %). Figure 2 shows scores for each dimension in the attitude domain, measured on a Likert scale. The majority (90 %) believed that hand hygiene is important. Overall, the attitude score did not differ between males and females (X^2^ = 3.822, df = 1, *p* = 0.051). Comparison of each dimension of the attitude domain against the knowledge category (< 50 % and ≥ 50 %) is given in the additional file as (Table S1). The participants with knowledge score three or more (≥ 50 %) had fewer negative attitudes such as “Hand hygiene is not important” (U = 4269, Z=-2.966, *p* = 0.003), “Do not agree with the recommendations or guidelines” (U = 4392, Z=-2.558, *p* = 0.011), and “My seniors or colleagues do not clean their hands, so do I” (U = 4363, Z=-2.622, *p* = 0.009).

#### Hand hygiene behavior

**Fig. 3:**
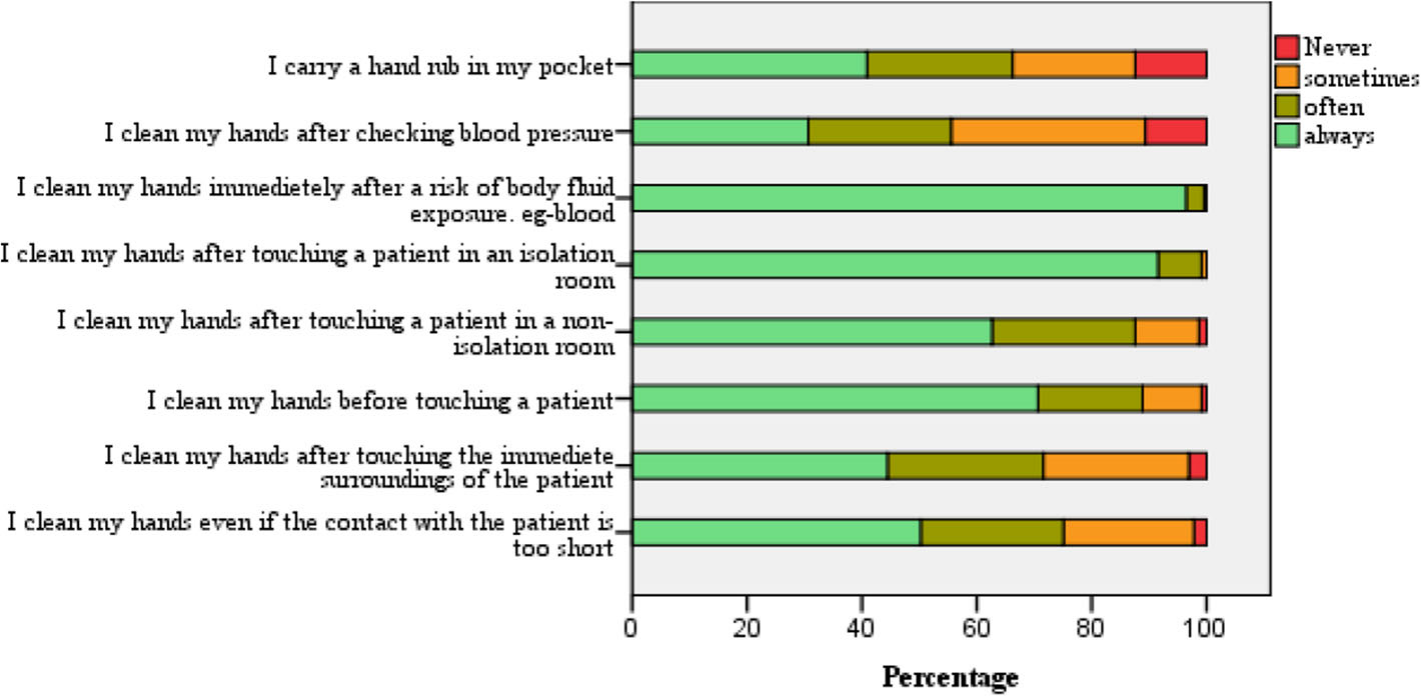
Participants’ behaviour on hand hygiene

The median score was 28 (range 16–32). However, only 36.4 % reported “adequate” hand hygiene performance in all eight dimensions of the behavior domain. Approximately 44.4 % did not clean their hands after checking blood pressure, 28.4 % after touching the patients’ immediate surroundings and 33.8 % did not carry a hand rub (Fig. 3). Almost all (> 99 %) cleaned their hands in high-risk circumstances; after touching a patient in isolation or with exposure to body fluids of the patient, but less so with low-risk conditions (viz. patients in the non-isolation area). Overall, reported hand hygiene behavior did not relate to either attitude score (*p* = 0.821) or knowledge score (*p* = 0.794) with bivariate correlation analysis. Each domain of reported hand hygiene behavior on the Likert scale was compared with knowledge (< 50 % and ≥ 50 %) and attitudes (positive vs. negative) using Mann Whitney U test. The results are provided in the additional file (Table S2 and S3). None of the reported behavior dimensions were significantly related to the knowledge score. Similarly, students’ attitudes (positive vs. negative) did not significantly relate to most reported behavior dimensions. However, students with more positive attitudes reported carrying a hand rub with them often or always (U = 4388, Z=-2.587, *p* = 0.010).

### Qualitative strand

Twelve participants were interviewed. Among them were seven females and five males. The mean age was 25.9 ± 1.03. Interviews lasted an average of 10 min (range: 9–11 min).

#### Motives for hand hygiene practices

The majority expressed personal and patient safety as advantages of hand hygiene practices, particularly during a pandemic. Some stated that the benefits are extended to the family members and friends [*The patients as well as us and … like our friends and family… all of us will benefit from hand hygiene*” (MS03)].

#### Negative and positive influencers of hand hygiene behavior

Participants described the important role the nursing staff played in hand hygiene practices [“… *nursing officers in particular, have reminded us to clean our hands and also seeing them practicing has reminded us to do it…”* (MS03)]. They also expressed the key role of the academic staff (senior physicians) as positive role models. Some reported that they adjusted their behavior to match the fellow students’ good practices. Besides, some junior staff members did not comply with hand hygiene guidelines and thereby the students were negatively influenced. Mixed opinions were expressed regarding patient influence. Some perceived that the patients are ignorant and did not disapprove of staff and students’ non-compliance, while others felt that patients were satisfied when staff adheres to hand hygiene practices [“*The patients in the ward that I am currently in… they don’t pay attention whether we wash hands or not… I think it’s not a good trend*” (MS-12)] and [“*I think they feel safe when they see us cleaning our hands…they are more supportive then…*” (MS05)].

#### Reasons for non-compliance

The main reason for non-compliance was adequate hand hygiene not being a habit [“*Probably because of the lack of practice …. maybe they forget as it has not been a routine before the pandemic… Just to change to a new practice within one or two months is difficult* (MS04)]. Most stressed that time constraints, workload, and overcrowded wards as obstacles [*It is because of the rush… and because of the heavy workload they tend to forget it* (MS10)]. Dryness and soreness of hands were emphasized as drawbacks, and a few expressed the notion that frequent hand washing could damage the normal flora in the skin, thereby leading to fungal infections. Also, they reported that lack of awareness was a reason for inadequate hand hygiene [*Most of the time I think it is their carelessness… and it is mostly seen among students.…. because we do not really see the depth of importance of hand hygiene* (MS07)]. Low availability and cost of hand sanitizer liquid and inconveniently placed hand washing facilities were also considered barriers.

#### Adjusting to a “new normal.“

The respondents were asked about the hand hygiene practices before and after the COVID-19 pandemic. Most stated that they hardly used alcohol hand rub before the pandemic in the wards and clinics [*“Before the pandemic the alcohol rubs were available… but the use was minimal. Only when we were at the theatre, yes… we used them”* (MS03)] and [“*Not before the pandemic I guess… but after the pandemic… yes, everyone is doing that* [cleaning hands]” (MS07)]. Others thought that the present pandemic had inculcated good habits [“*Now I wash my hands quite often, just like what WHO has told us to do, this pandemic in fact has taught us a lesson*…” (MS11)].

#### Education and supervision

The participants made several suggestions about how to increase knowledge and awareness. They preferred practical demonstrations in addition to lectures. Also, they praised the hospital circulars and media contribution reinforcing hand hygiene practices. The participants highlighted the need for adequate supervision and feedback and reminders for hand hygiene [“*When we enter a shop, the doorman remind us to clean our hands…. but in the hospital, there is no one to tell us … obviously, at times…. we forget”* (MS04)]. They also stressed the importance of ward staff being role models to influence their hand hygiene practices.

## Discussion

The present study focused on knowledge, attitudes, and reported behavior related to hand hygiene of undergraduate medical students during their clinical placements in the final year. The mixed-method design of our study allowed for more specific and detailed information on hand hygiene practices and to explore the hand hygiene behavior before the pandemic. It appeared that a higher fraction of participants had positive attitudes towards hand hygiene. Their knowledge and reported compliance were comparatively lower than the attitudes. Thus, an exploration of strategies to bridge this knowledge-attitude-behavior gap is needed.

### Knowledge and attitudes

In 2016, Ariyaratne et al. reported that 17 % of Sri Lankan medical students having poor knowledge compared to 31.6 % in the present study [9]. Similar studies reported from developed countries had shown better knowledge scores. A study from Spain reported a mean knowledge score of 4.59 out of six (compared to 3.35 out of six in our study), with a lower percentage of respondents (4.69 %) scoring three or less out of six and a higher percentage (80 %) scoring more than four [10]. However, the comparison between different studies is somewhat contentious. Variations in study questionnaires and settings impact comparisons and undermine the generalizability of the findings.

 The participants scored noticeably low on the correct procedure of hand hygiene using alcohol hand rub, tested on many dimensions such as drying time and concentration of alcohol. Appropriate alcohol concentration and drying time are the primary drivers of efficacy [11]. It is essential to understand that efficacy also depends on the alcohol quantity applied.

Participants with higher knowledge had fewer negative attitudes. Most believed that hand hygiene was as important as treating patients and agreed with the current recommendations and guidelines. They also cited that, in general, attitudes had improved more than that was before the pandemic. In contrast, in the qualitative strand, the participants also highlighted a few negative attitudes. Several of them reflected that non-compliance was related to negligence and not realizing the importance.

Notably, among our participants, unlike knowledge, attitudes were good. Ariyaratne et al., in 2016, described poor attitudes compared to knowledge from a similar cohort in Sri Lanka [9]. The likely explanation for better attitudes among our study participants could be related to risk aversion intention during the pandemic. We also observed that participants with higher knowledge scores had fewer negative attitudes. Therefore, it is likely that more emphasis on knowledge increase will improve attitudes further.

### Hand hygiene behavior

Among the different hand hygiene moments, higher reported compliance was observed before and after direct patient contact, particularly with patients in the isolation area. Yet, reported compliance was noticeably low after contact with patient surroundings or short patient contact time. Many studies have shown that high-touch surfaces (viz. bed rail, blood pressure cuff, bedside table, intravenous pump, and bed surface) are reservoirs of pathogens leading to cross-contamination [12]. Pathogenic micro-organisms are generally collected over a long time as they can survive for hours to months on surfaces and medical equipment [13].

Previously, positive attitudes have been reported to be significantly associated with the hand hygiene behavior of nurses and physicians [14, 15]. In contrast, in our study, attitudes did not impact the reported behavior domain significantly. However, in the participants’ interviews, many cited a lack of habit as a reason for non-compliance. They expressed that they tend to forget as the practice of hand hygiene is not habitual. Habit is a phenomenon in which individuals, rather than focusing on new information, fall back on previous automatic responses [16]. Thus, despite our study participants having better intentions, they probably maintained their previous behavior. They cited that frequent reminders would enhance their practices. Therefore, cues (verbal or visual) could probably prompt correct behavior and, in turn, develop into a habit. Habits should thus persist even when conscious motivation wanes [16].

### Strategies to improve hand hygiene compliance and competence

Based on the findings of the present study, we suggest several strategies to improve hand hygiene competence among medical students during clinical placements. In that context, most importantly, the knowledge-attitude-behavior gap could be addressed by empowering them with evidence on health benefits and reinforcement of positive behavior. Role modeling, reminders, hospital protocols, and supervision could enhance acceptable behavior. Senior academic staff supervision and adherence to hand hygiene was a stimulus to the students in our study. Also, the participants valued non-hierarchical role models (e.g., peers). Therefore, positive role models in the clinical setting are an essential component that should be emphasized in the training curriculum [17]. This helps them to build up enthusiasm for behavior adjustments, probably more than any other learning experience.

Also, a habitual mindset for hand hygiene needs to be developed to repeat actions [18]. Verbal cues, as well as poster displays, could act as reminders to sustain hand hygiene compliance. Feather et al. observed the handwashing behavior of 187 final-year medical students at a clinical station during an OSCE exam [19]. Only 8.5 % cleaned hands after patient contact, which improved to 18.3 % when a handwashing sign was displayed.

Scientific knowledge is also an important component to inculcate correct hand hygiene measures although, educational programs alone are inadequate for long-lasting improvement [20]. Students should be aware that alcohol hand sanitizers are an effective alternative to handwashing, although their efficacy is generally lower against non-enveloped viruses [21]. Dry time, type of alcohol, and amount are important aspects that the students need to know. Smaller volumes of hand rub and short dry time do not ensure efficacy [22]. Since the drying time is longer with alcohol-based products, health care workers are likely to use insufficient volumes to hasten the drying time, and therefore do not use adequate amounts for cleansing [23]. As suggested by the participants, practical clinical sessions may help them experience hands-on sessions, which would increase good patient-safety habits and knowledge. In a previous study, > 90 % of students rated the practical sessions using the ultra-violet light hand inspection cabinet as an effective teaching method [24]. Knowledge dissemination could also be carried out through lectures and tutorial sessions.

On the other hand, the elimination of barriers to hand hygiene needs to be addressed. Time constraints, skin irritation, workload, and non-availability of alcohol hand rub were mentioned as barriers to non-compliance in our cohort. Handwashing with soap and water, which is freely available in almost all facilities, is an alternative to alcohol hand rub in resource-poor settings. Also, moisturizing cream should be encouraged to lessen skin dryness and irritation [25].

The participants had perceived a notable improvement in hand hygiene compliance among staff and students during the pandemic compared to the pre-pandemic period. Thus, enhancing positive behavior through educational interventions would lead to habitual engagement in hand hygiene, which is likely to persist even in the post-pandemic period.

### Limitations

The study results should be interpreted with the following limitations. A qualitative survey was carried out in only one training center. In the quantitative and qualitative survey, participants were not enrolled using systematic random sampling. Therefore, the sample may have had a selection bias affecting the generalizability of the findings. The lack of a validated tool for knowledge assessment and missing control group to compare data before the Covid-19 pandemic was identified as limitations of the study. Also, we did not measure hand hygiene behavior objectively; thus, it may have overestimated the actual compliance in real-life practice.

## Conclusions

Most of the participants had positive attitudes towards hand hygiene. Yet, a considerable gap between attitudes and knowledge and reported hand hygiene behavior was evident. Coupling educational programs that use cognitive and behavioral methods, including role modeling, supervision, and frequent reminders, is recommended to bridge the knowledge-attitude-behavior gap.

## Supplementary information

**Additional file 1.**

## Data Availability

The data of this study are not publicly accessible due to ethical restrictions of the Local Ethics Committee. However, access could be made available from the corresponding author on a reasonable request after obtaining approval from the Ethics Committee.

## Abbreviations

COVID-19: Coronavirus Disease 2019
MS: Medical Student
WHO: World Health Organization
