# Supplementary material for: Hand hygiene behavior among Sri Lankan medical students during COVID-19 pandemic
Source: BMC Med Educ. 2021 Jun 8;21:333. doi: 10.1186/s12909-021-02783-9 (PMC8186824; doi:10.1186/s12909-021-02783-9)
Supplement: Supplementary file 1 — Additional file 1. [file 12909_2021_2783_MOESM1_ESM.docx]

Table S1: Comparison of dimensions of the attitude domain with knowledge category

|  | | | | |
| --- | --- | --- | --- | --- |
|  | I feel that patient care needs priority than spending time on hand cleaning | My seniors or colleagues do not clean their hands, so do I | I do not think hand hygiene is very important | I do not agree with the recommendations or guidelines |
| Mann-Whitney U | 4889.500 | 4363.000 | 4269.500 | 4392.000 |
| Wilcoxon W | 7445.500 | 6919.000 | 6825.500 | 6948.000 |
| Z | -1.366 | -2.622 | -2.966 | -2.558 |
| Asymp. Sig. (2-tailed) | .172 | .009 | .003 | .011 |
| Grouping variable: Knowledge <50% and ≥50% | | | | |
|  | |  |  |  |

Table S2: : Comparison of dimensions of the behavior domain with knowledge category

|  | | | | | | | | |
| --- | --- | --- | --- | --- | --- | --- | --- | --- |
|  | I clean my hands before touching a patient | I clean my hands When the contact with the patient is too short | I clean my hands after touching the immediate surroundings of the patient | I clean my hands after touching a patient in a non-isolation room | I clean my hands after touching a patient in an isolation room | I clean my hands immediately after a risk of body fluid exposure. eg-blood | I clean my hands after checking blood pressure | I carry a hand rub in my pocket |
| Mann-Whitney U | 5048.000 | 5020.000 | 5290.000 | 4990.000 | 5127.000 | 5295.000 | 5043.000 | 5367.000 |
| Wilcoxon W | 16983.000 | 16955.000 | 17225.000 | 16925.000 | 17062.000 | 17230.000 | 16978.000 | 7923.000 |
| Z | -1.154 | -1.071 | -.417 | -1.224 | -1.555 | -1.182 | -.976 | -.232 |
| Asymp. Sig. (2-tailed) | .248 | .284 | .677 | .221 | .120 | .237 | .329 | .817 |
| Grouping variable: Knowledge <50% and ≥50% | | | | | | | | |

Table S3: : Comparison of dimensions of the behavior domain with attitude category

|  | | | | | | | | |
| --- | --- | --- | --- | --- | --- | --- | --- | --- |
|  | I clean my hands before touching a patient | I clean my hands When the contact with the patient is too short | I clean my hands after touching the immediate surroundings of the patient | I clean my hands after touching a patient in a non-isolation room | I clean my hands after touching a patient in an isolation room | I clean my hands immediately after a risk of body fluid exposure. eg-blood | I clean my hands after checking blood pressure | I carry a hand rub in my pocket |
| Mann-Whitney U | 5160.000 | 5279.000 | 5230.500 | 5242.500 | 5411.500 | 5348.000 | 5181.000 | 4388.000 |
| Wilcoxon W | 7788.000 | 7907.000 | 17011.500 | 7870.500 | 8039.500 | 7976.000 | 16962.000 | 7016.000 |
| Z | -.955 | -.547 | -.651 | -.679 | -.440 | -1.095 | -.750 | -2.587 |
| Asymp. Sig. (2-tailed) | .340 | .585 | .515 | .497 | .660 | .273 | .453 | .010 |
| Grouping Variable: Attitudes: ≥80% and <80% | | | | | | | | |
